# Spatial Genetic Structure in Natural Populations of *Phragmites australis* in a Mosaic of Saline Habitats in the Yellow River Delta, China

**DOI:** 10.1371/journal.pone.0043334

**Published:** 2012-08-16

**Authors:** Lexuan Gao, Shaoqing Tang, Liqiong Zhuge, Ming Nie, Zhu Zhu, Bo Li, Ji Yang

**Affiliations:** 1 Center for Evolutionary Biology and Institute of Biodiversity Science, Fudan University, Shanghai, China; 2 College of Life Sciences, Guangxi Normal University, Guilin, China; 3 Center for Watershed Ecology, Nanchang University, Nanchang, China; CNR, Italy

## Abstract

Determination of spatial genetic structure (SGS) in natural populations is important for both theoretical aspects of evolutionary genetics and their application in species conservation and ecological restoration. In this study, we examined genetic diversity within and among the natural populations of a cosmopolitan grass *Phragmites australis* (common reed) in the Yellow River Delta (YRD), China, where a mosaic of habitat patches varying in soil salinity was detected. We demonstrated that, despite their close geographic proximity, the common reed populations in the YRD significantly diverged at six microsatellite loci, exhibiting a strong association of genetic variation with habitat heterogeneity. Genetic distances among populations were best explained as a function of environmental difference, rather than geographical distance. Although the level of genetic divergence among populations was relatively low (*F*’_ST_ = 0.073), weak but significant genetic differentiation, as well as the concordance between ecological and genetic landscapes, suggests spatial structuring of genotypes in relation to patchy habitats. These findings not only provided insights into the population dynamics of common reed in changing environments, but also demonstrated the feasibility of using habitat patches in a mosaic landscape as test systems to identify appropriate genetic sources for ecological restoration.

## Introduction

Spatial configuration of suitable environments for organisms is often patchily structured at various scales, from biogeographic regions to local environments within a landscape [1], [2], [3], [4]. Diverse patches of habitat created by natural disturbances have profound influences on ecological and evolutionary processes across scales [2], [4], [5], [6], [7], [8], [9]. The population structure and dynamics of species in patchy environments depend on the spatial arrangement and heterogeneity of habitats [2], [8], [9], [10], [11], [12], [13], [14], [15].

Numerous studies have revealed the role of environmental patchiness in generating spatial genetic structure (SGS) in natural populations [10], [16], [17], [18], [19], [20], [21], [22], [23]. SGS, i.e. non-random spatial distribution of genotypes and alleles, can result from different processes, including restricted gene dispersal, genetic drift, and micro-environmental selection [22], [24], [25]. Understanding the processes underlying population structure and its relationships with habitat structure and heterogeneity can not only help reveal the potential driving forces promoting population divergence and adaptation, but may also contribute to the prediction of how populations will respond to changing environments, which is important for both conservation and restoration efforts.

The Yellow River Delta (YRD) lies in the eastern coastal area of China, which is formed by sediment deposition of the Yellow River [26]. Due to on-going aggradation, the delta at the mouth of the river is still expanding at about 20 km^2^ of new land created each year [27], [28], [29]. The YRD has a monsoon climate of the warm-temperate zone with mean annual temperature of 11.7–12.6°C. The mean annual precipitation is 530–630 mm, while the mean annual evaporation is 1750–2430 mm [30]. The excess evaporation from soil, together with seawater encroachment, has led to serious soil salinization in this region [31], [32]. The lowland reaches of the Yellow River are highly unstable, which has changed its route more than 10 times since 1855 [33]. The course shifts of the lower Yellow River not only gave rise to the formation of new delta lobes along the coast, but also significantly influenced the depth of ground water table and the quality of ground water (i.e. proportion of salty to fresh water) via lateral seepage in areas at both sides of the river channel. As the result of the joint effect of Yellow River lateral seepage and seawater intrusion, a mosaic of contrasting environments that differ in soil salinity was generated in the YRD region, which exerts profound influence on the growth and adaptation of organisms living in this area [34], [35].

Common reed, *Phragmites australis*, is one of the dominant species in the YRD region, acting as an ecosystem engineer in the restoration of the YRD ecosystem [36]. This species is not a true halophyte, but it seems well adapted to the heterogeneous environments in the YRD and can tolerate wide range of soil salinity [37], [38]. Population genetic patterns of common reed have been investigated at different scales [39], [40], [41], [42], [43], [44], [45], [46], [47]. Salt tolerance of the reed has also been assessed in several studies [48], [49], [50], [51]. However, previous studies were mostly focused on detection of genetic relationships between populations distributed across different ecological and geographic areas, to explore historical events of long-distance migration and invasion. Few studies have sampled extensively from adjacent sites to specifically examine micro-geographic genetic structure. Less is known about how contemporary factors such as habitat heterogeneity impact local population structure and dynamics in common reed.

**Figure 1:**
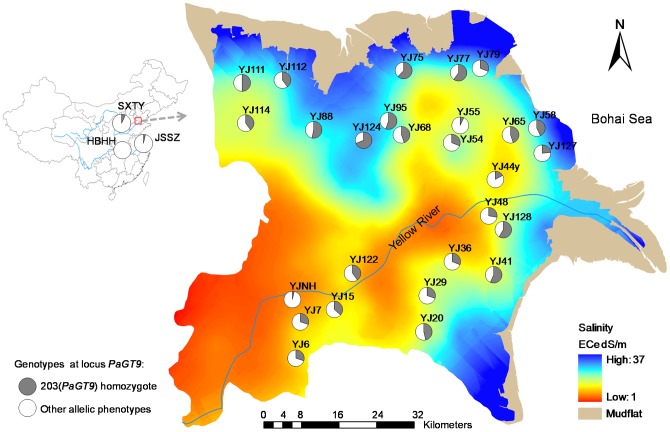
Schematic map showing sampling localities and the tendency of soil salinity based on OK predictions. Pie graphs show the frequency distribution of the 203(*PaGT9*) homozygote and other allelic phenotypes in different populations.

Populations occupying adjacent but contrasting habitats in the YRD present distinct advantages for assessing the effects of spatial environmental heterogeneity in relation to soil salinity on population genetic dynamics of the reed. In this study, we examined the level of genetic variation within and among the reed populations in the YRD, using microsatellite (also known as Simple Sequence Repeat, SSR) markers. By exploring the correlation between genetic diversity and environmental heterogeneity, we sought to determine whether patchy habitats have structured patchily distributed populations in this area, to assess the effect of the spatial arrangement of habitat patches on the genetic structure of common reed in the YRD. We also aimed to address the feasibility of using patchy habitats as test systems to evaluate the potential for plants to adapt dynamically to changing environments, which is vitally important for selecting appropriate plant materials for ecological restoration [52].

**Table 1:** Sampling sites of the common reed in Yellow River Delta.

Site	Samplesize	Longitude	Latitude	Soil salinityECe (dS/m)
YJNH	29	118.39256	37.60591	4.52*
YJ44y	30	118.75068	37.74607	6.38*
YJ6	30	118.40399	37.47777	7.12*
YJ7	30	118.39628	37.53886	7.31*
YJ48	29	118.87539	37.74002	8.19**
YJ122	30	118.54735	37.63576	8.76**
YJ15	30	118.50631	37.56479	8.99**
YJ65	29	118.94426	37.88558	9.69**
YJ128	29	118.90295	37.71011	10.96**
YJ29	30	118.72592	37.58559	11.15**
YJ36	29	118.78371	37.64583	11.37**
YJ114	30	118.27461	37.92928	11.85**
YJ20	28	118.71681	37.51787	11.97**
YJ41	30	118.88227	37.62161	12.15**
YJ111	28	118.28079	38.00467	12.74**
YJ55	29	118.81202	37.90436	12.81**
YJ77	30	118.80450	38.01746	12.86**
YJ54	29	118.79557	37.87476	13.74**
YJ127	30	119.05893	37.86028	13.80**
YJ68	29	118.67387	37.89626	14.05**
YJ79	29	118.86720	38.01841	15.00**
YJ112	30	118.40862	38.00918	15.12**
YJ58	28	118.99667	37.90446	15.30**
YJ88	30	118.45609	37.91095	15.88**
YJ75	29	118.68672	38.01987	17.09***
YJ95	29	118.64481	37.91907	18.05***
YJ124	29	118.57326	37.88456	20.12***

**Soil salinity class:**

* slightly saline;

** mediately saline;

*** highly saline.

## Materials and Methods

### Study Area and Sampling

This study was conducted over an area of approximately 6622 km^2^ in the YRD (Fig. 1). Local-scale variation of soil salinity was assessed based on a square grid sampling design [53]. A total of 219 points were systematically selected at the centers of 5 km square grids, each of which corresponded to one sample with 25 km^2^. At each site 3–6 soil samples were collected. The soil samples were taken to the laboratory for measuring saturated paste conductivity (ECe) by using 1/5 diluted extracts (the electrical conductivity (EC) of 1∶5 soil-water extract) [54]. The distribution map of soil salinity (ECe) was created using the ordinary kriging (OK) interpolation technique. According to the soil salinity classes proposed by Richards [55], ECe ranges of <4, 4–8, 8–16, 16–32 and >32 dS/m corresponded to non-saline, slightly saline, mediately saline, highly saline and extremely saline, respectively [54].

**Table 2:** Primer sequences and allelic diversity information for six microsatellite loci of the common reed.

Locus	Primer sequences (5′–3′)	Maximum no.alleles per sample	Allele sizerange (bp)	Total no.alleles	Total no. allelephenotypes
*PaGT4*	F: TGCTCCCTGCCAGTTTCTTG	4	266–278	5	23
	R: TATCCACCCTTCGAAGGCAC				
*PaGT8*	F: TCTGAACATAATCCTGGTGG	4	171–191	7	39
	R: TCTGTGTGAAGCAGTTCTGC				
*PaGT9*	F: CCATGTGTTAATGTTGTCC	4	187–213	11	102
	R: ATTGAATCCACACGTTTCCG				
*PaGT11*	F: CAACTCCGTGAATGACATGC	4	141–151	6	19
	R: CAGTTTGTGCACTAATGGAC				
*PaGT14*	F: GTTGCAGCAAGTATTTGG	5	166–222	26	228
	R: CAAGCATTCTAGTAGTAGC				
*PaGT16*	F: ACCAATCAGTCAGACTAGCC	3	232–292	11	39
	R: GTTCTCATGTTGGAGAAGCC				

Since the YRD has been exploited on a large scale, with many areas in this ecoregion being converted to agricultural or other land uses, a total of 27 sites across the YRD region, with relatively little human disturbance and more than 30 visibly separated patches of common reed, were selected for sampling. The location information and soil salinity of each site are shown in Table 1. The common reed can reproduce both clonally through rhizome growth and sexually through wind-pollinated seeds. To decrease the likelihood of sampling clone-mates, each sample (28 to 30 per population) was collected from distinct patches that were separated by a minimum distance of 3 m at each site. Three additional non-saline sites surrounding the YRD: SXTY (E112°33′ N37°52′), HBHH (E113°28′ N29°48′) and JSSZ (E120°35′ N31°17′), were also included in the study for collecting plant materials for comparative analysis. For each population, 28–30 leaf samples were collected and stored in silica gel until DNA extraction and genotyping.

**Figure 2:**
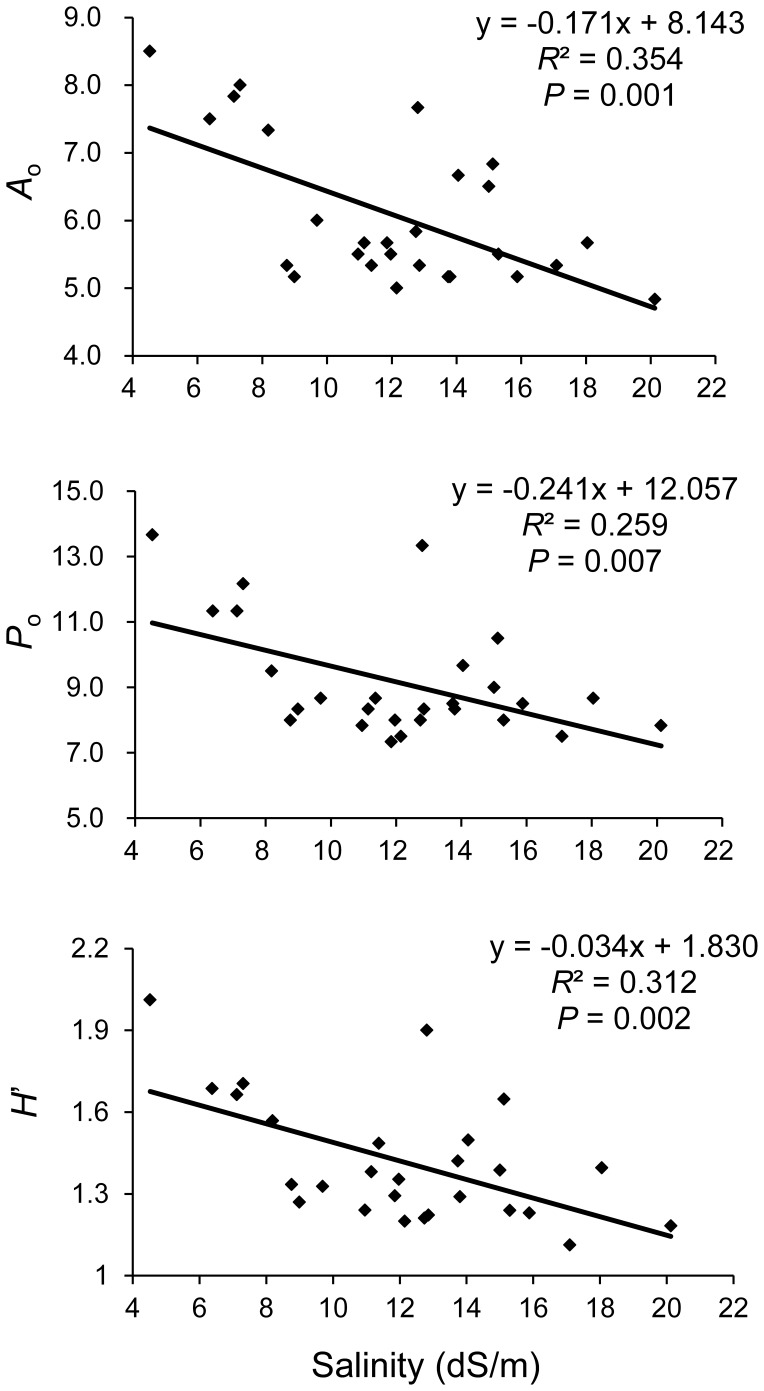
Correlation between soil salinity and population genetic diversity in YRD. Values of the observed number of alleles (*A*_o_), allelic phenotypes (*P*_o_), and the allele phenotype diversity statistic (*H’*) of each population are mean values over six microsatellite loci.

### SSR Analysis

Total genomic DNA was extracted following the CTAB extraction protocol [56]. Six microsatellite loci (PaGT4, PaGT8, PaGT9, PaGT11, PaGT14 and PaGT16) were used to assess the genetic diversity and structure of 30 common reed populations, using primers developed by Saltonstall [45] (Table 2). The PCR reactions were carried out in a total volume of 20 µl containing 1×PCR buffer, 0.2 mM dNTPs, 6 pmol of each forward and reverse primer, 20 ng of genomic DNA, and 0.5 U Taq polymerase (TAKARA). Reactions were run in an Eppendorf Mastercycler using the following program: an initial denaturation at 94°C for 6 min, followed by 35 cycles of 94°C for 30 s, 53–58°C for 30 s and 72°C for 4 s, and a final extension at 72°C for 2 min. The amplified products were resolved on 6% polyacrylamide sequencing gels and silver stained according to the protocol described by Bassam et al. [57].

**Figure 3:**
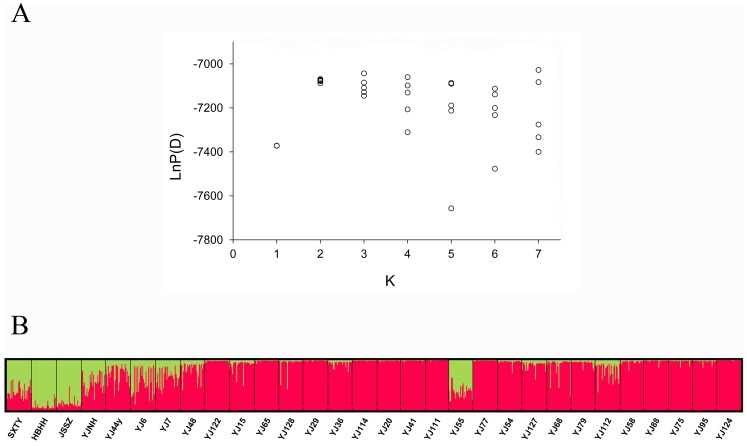
Estimated population structure for common reed from STRUCTURE analysis. (A) Plot of the log probability of the data [LnP(D)] given values for K from 1 to 7. Circles represent the likelihood values of 5 replicate runs at each K value. (B) Population clustering for K = 2. Each individual is represented by a thin vertical line, which is partitioned into K coloured segments that represent the individual’s estimated membership fractions. Black lines separate individuals from different sampling sites, which are labelled below the figure.

### Analysis of Genetic Diversity

Due to the polyploid nature of the common reed, several microsatellite DNA alleles might simultaneously occur at a single locus. Even if only two alleles were detected at a single locus, the allelic configurations might also be greatly variable among individuals as each allele can be present in more than one copy. Considering the difficulties in assessing the actual genotype of each individual based on band intensity and accurately estimating the number of copies of an allele in heterozygous individuals, the banding patterns observed at each polymorphic locus were recorded as ‘allelic phenotypes’ [45], [58] in this study. Each phenotype scored alleles as present or absent, regardless of allele dosage (allele frequency). This may result in underestimation of genetic variation present but avoids overestimating genetic diversity as a result of incorrectly calculating the number of alleles [59]. For each polymorphic locus, the number of alleles (*A*_o_) and allelic phenotypes (*P*_o_) were counted. We compared multilocus allele phenotypes found within and among populations to identify samples with identical genetic phenotypes. Repeated phenotypes within populations were assumed to result from asexual reproduction (ramets of a single genet.) and thus excluded from analyses of genetic diversity and differentiation because repeated sampling of a single clonal individual can unduly influence estimates of the distribution of genetic variation [59]. Two genetic-differentiation statistics based on allelic phenotype data were calculated using the program F-DASH (1000 permutations) [60]. Within-population genetic diversity was estimated using a simple allele phenotype diversity statistic (*H’*) based on the average number of unshared alleles between pairs of individuals. Genetic differences between populations were measured with *F’*_ST_ based on the proportion of genetic variance among populations relative to the total genetic diversity. The Bayesian method, implemented in HICKORY version 1.1 [61], was also used to estimate the heterozygosity within each population (*h*_s_) and the level of genetic differentiation among populations (*θ* ^II^). Computations were carried out using the default values, as recommended in the manual [61].

**Figure 4:**
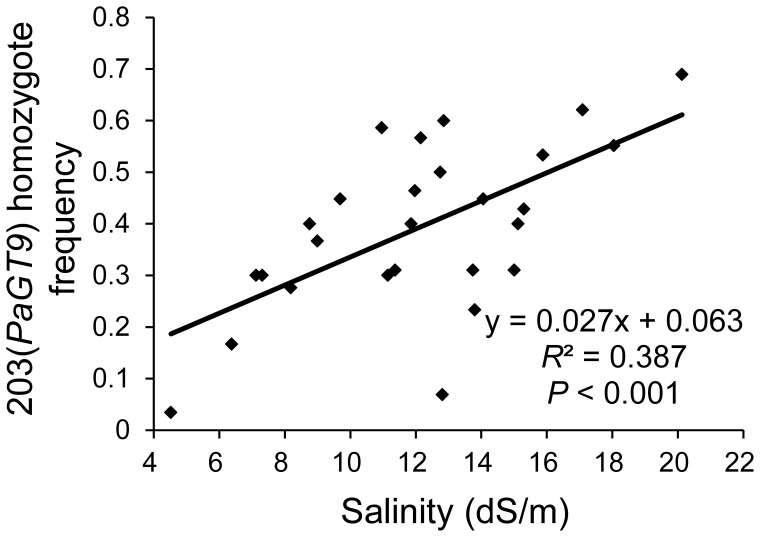
Correlation between the frequency of the 203(*PaGT9*) homozygote and soil salinity, showing the increasing tendency of the 203(*PaGT9*) homozygote in populations inhabiting saline habitats in YRD.

### Testing Effects of Ecogeographic Variables on Genetic Variation

Several complementary approaches were used to examine the correlations between genetic patterns, geographic distances, and soil property (salinity). We first examined the effects of geographical location and habitat type on the levels of genetic variation in each population by regression analysis of genetic diversity characteristics (number of alleles, allelic phenotypes and allele phenotype diversity statistics) and their associations with latitude, longitude and soil salinity using SigmaStat 3.5 (Systat Software, Inc., CA, USA).

We then tested the correlation between genetic and geographic distances using the Mantel test [62], to investigate if the genetic differentiation in reed populations was independent of geographic distances. Population genetic distances were calculated using the program POPDIST [63]. Mantel tests were conducted using the NTSYSpc 2.0 software [64]. Significance was determined using 1000 permutations. The Bayesian model-based clustering method [65] implemented in STRUCTURE v2.2 [66], [67], [68] was also used to investigate population structure and infer the most likely number of populations in our sample. In this approach, multilocus genotypic data were used to define a set of populations with distinct allele frequencies and to assign individuals probabilistically to them. We ran the analysis for up to 7 possible populations (*K* = 1–7) using the admixture model with correlated allele frequencies. We performed 5 independent runs for each *K* value with 100 000 burn-ins and 100 000 iterations after burn-ins. The number of population groups best fitting our data set was defined by Δ*K* as suggested by Evanno et al. [69].

Finally, we conducted a spatial analysis to estimate the association between each allele and soil salinity using the program SAM [70], [71]. This program works at the individual level and inspects correlations between each allele and the soil salinity by univariate logistic regression [71], [72]. In this analysis, we recorded our data in a dominant way, with each allele at each locus coded as present or absent. The analysis thus directly links allele occurrence with environmental variables. Both statistical tests, the likelihood ratio test and the Wald test implemented in SAM, were used to assess the significance of coefficients calculated by the logistic regression model to ensure the robustness of the method.

## Results

A total of 66 alleles were detected in 882 common reed individuals for the six polymorphic microsatellite loci. The number of alleles scored at each locus (*A*_o_) varied from 5 (*PaGT4*) to 26 (*PaGT14*), with an average of 11 alleles. The number of allele phenotypes at a single locus (*P*_o_) ranged between 19 (*PaGT11*) and 228 (*PaGT14*) (Table 2). Two individuals from the population YJ20 were found to share the same multilocus allele phenotype, and one of them was thus excluded from the data matrix. The values of *A*_o_ and *P*_o_ for each population are shown in Table S1. Genetic diversity within populations (*H’*) ranged from 1.11 to 2.01 (Table S1). The overall population differentiation was *F’*_ST_ = 0.073.

Significant negative correlations were detected between soil salinity and mean observed number of alleles (*A*_o_) (*R* ^2^ = 0.354, *P* = 0.001), the observed number of allele phenotypes (*P*_o_) (*R* ^2^ = 0.259, *P* = 0.007), and the within-population genetic diversity statistics (*H’*) (*R* ^2^ = 0.312, *P* = 0.002) over six microsatellite loci (Fig. 2), suggesting a progressive reduction in genetic diversity along with increasing soil salinity in reed populations. The Bayesian estimates for within-population genetic diversity exhibited a similar negative correlation with soil salinity (*R* ^2^ = 0.599, *P*<0.001) (Fig. S1). We also compared the allelic diversity among population groups occupying non-saline, slightly saline, and highly saline habitats, respectively. The results showed that the values of *A*_o_, *P*_o_, and *H’* of populations from non- and lower saline environments were clearly higher than those of populations from highly saline habitats (Fig. S2, Table S2), which were consistent with the results of correlation analyses. However, the Mantel test demonstrated no significant correlation between genetic and spatial distances of different populations (*P* = 0.57) (Table S3), indicating that the genetic differentiation in reed populations was independent of the geographic distance.

According to Ln*P*(*D*) and Δ*K* values, two clusters (*K* = 2) best fit the dataset, indicating that two genetically distinctive population groups existed in common reed populations. The clustering of individuals into two groups based on genetic landscapes suggested a division of sample sites into saline and non-saline habitats. Grouping of individuals by geographical location (populations), however, showed high levels of population admixture, as most populations consisted of individuals from different clusters (Fig. 3).

Using the program SAM, we tested for the association between allelic frequency and soil salinity in natural populations of common reed, and identified 3 alleles as significant with both Wald and likelihood ratio G tests, with a significance level being 5.05 e^−06^ (corresponding to a 99% confidence level including Bonferroni correction) (Table S4). Because of the polyploid nature of the common reed, it was difficult to get an accurate estimation of the frequencies of these alleles in natural populations. Instead, we calculated the frequencies of heterozygous and homozygous individuals with these alleles in different populations, and found a significant positive correlation between the 203(*PaGT9*) frequency and the soil salinity of sampling sites, with the frequency of the 203(*PaGT9*) homozygote increasing from 3.4% for the lowest saline population (YJNH) to 69.0% for the highest saline population (YJ124) (*R* ^2^ = 0.387, *P*<0.001) (Fig. 4).

## Discussion

Despite their close geographic proximity, the common reed populations in the YRD significantly diverged in overall genetic diversity within population, and in allele frequencies at six microsatellite loci. The YRD common reed populations exhibited a strong association of genetic variation with environmental heterogeneity, i.e. the salinity differences of different habitats, independent of geographical distance. Although the level of genetic divergence among populations was relatively low (*F*’_ST_ = 0.073), weak but significant genetic differentiation, as well as the concordance between ecological and genetic landscapes, suggested some degree of population structuring of the common reed in the YRD.

Several alternative scenarios may explain the pattern of genetic variation observed in common reed. First, patchy habitats might have restricted pollen and seed dispersal, leading to spatial genetic structure [73]. Second, past colonization events and/or recent range expansions were accompanied by founder effects and genetic drift, resulting in an accumulation of rare alleles and a reduction in genetic diversity in local populations [74], [75]. Finally, distinct environmental optima affecting fitness caused structure as populations diverged [8], [24], [76]. By comparing the allelic diversity of different reed populations, we found that most frequent alleles were shared among populations. Subsequent STRUCTURE analysis further suggested the high levels of population admixture, indicating a common source population for the reed populations in the YRD or extensive gene flow/migration among populations. Limited dispersal thus seems not to be a likely scenario leading to the spatial genetic structure in the reed populations in the YRD.

The Yellow River Delta is an ecoregion with heterogeneous and changing environments. Organisms living in this area have been subject to spatial and temporal variation in habitat suitability. To track the changing distribution of suitable environments, range expansion through frequent colonization of new sites has occurred recurrently in the history of most species. Strong genetic drift may be associated with range expansions, generating genetic patterns in allele frequencies that are quite different from what is expected in equilibrium populations [74]. Genetic diversity can be reduced in expanding populations as only a few individuals contribute genetic variation to the newly colonized populations [75], [77]. In contrast, some alleles may reach a high frequency because of repeated founder events [78], a process called genetic surfing [79]. Genetic differentiation may thus be produced among newly formed populations by intense genetic drift during population expansion [75], [79], [80]. In this study, we found that some allele phenotypes were shared among populations. Most populations were admixed and consisted of allele phenotypes from multiple populations, probably suggesting multiple founder events. Decline in genetic diversity was also detected in some populations, with the frequency of a specific allele increasing significantly. We thus cannot rule out the role for demographic history in shaping the genetic differences between reed populations. However, the strong concordance between patterns of genetic and ecological variations also dropped a hint of the potential role of habitat heterogeneity in driving population divergence.

Spatial variation in the environment may affect genetic variation in populations living there [76]. Population dynamics of patchily distributed species are affected by density-independent environmental fluctuations [81]. The genetic structure of populations is not always reflected in the geographical proximity of individuals, and individuals with different geographical locations are not necessarily genetically differentiated [69]. We showed in this study that, within the environmental mosaics in the YRD, the common reed populations displayed a mosaic pattern of genetic differentiation. The overall genetic diversity within populations decreased progressively along with the increasing soil salinity. The locus *PaGT9* exhibited an elevated variation among populations exceeding neutral expectations, with the allele 203(*PaGT9*) showing a significantly higher frequency in saline habitats than in other sites. The genetic distances between reed populations in the YRD were best explained as a function of environmental differences, rather than geographical distance. Both reduction in genetic diversity and the increase in allele frequency could arise as results of chance for sexual reproduction involving a limited number of genotypes during range expansion and subsequent limitation in seed dispersal. However, these patterns of genetic variation not only occurred at several separate sites in the YRD, but also covariated with environmental salinity. In addition, the large population sizes of common reed at our study sites and the high levels of gene flow among populations should greatly reduce the likelihood of genetic surfing during range expansion. Therefore, genetic divergence among reed populations in the YRD is unlikely to be driven by strictly neutral processes. The correspondence between ecological and genetic landscapes may be indicative of the potential role of environmental variables in driving population divergence [82], [83], [84], [85].

Correlations between phenotype and environment may be mirrored at the level of individual genetic polymorphisms. A powerful way to detect the footprint of selection in natural situations is to associate allele frequency with environmental variations. A number of previous studies have shown that there is a correlation between genetic diversity and environmental heterogeneity in common reed populations [86], [87], [88], [89], but very few studies have explicitly tested the causal environmental factors behind the pattern of genetic variation. This situation is largely due to the fact that environmental heterogeneities were mostly ill-defined in the earlier studies, leading to the uncertainty in selective agent predictions. Spatial analysis of population genetic structure in mosaic environments, from the perspective of landscape genetics, can not only help reveal the potential of dynamic adaptation of organisms to changing environments, but may also contribute to the identification of environmental factors that structure intraspecific genetic diversity, which are of interest for both conservation and ecological restoration. Although such an analysis may just represent the first step in the study of local adaptation, and the results alone are merely correlative, not *de facto* evidence of adaptive differentiation, it is efficient to identify subtle population structure and concomitant environmental variation representative of a potential selection gradient, to facilitate a further analysis and test of adaptation [90]. In conclusion, our findings not only provided insights into the population dynamics of common reed in changing environments, but also demonstrated the feasibility of using habitat patches in a mosaic landscape as test systems to identify appropriate genetic sources for ecological restoration.

## Supporting Information

Figure S1 **Correlation between soil salinity and the heterozygosity of each population (** ***h*** **_s_) estimated with Hickory v1.1.**
(TIF)

Figure S2 **Comparisons of genetic diversity among the common reed populations from different salinity environments.** *A*_o_: observed number of alleles; *P*_o_: observed number of allele phenotypes; *H*’: allele phenotype diversity statistic based on the average number of unshared alleles between pairs of individuals.
(TIF)

Table S1 **Genetic characteristics of the common reed populations from YRD based on six microsatellite loci.**
(XLSX)

Table S2 **Comparisons of genetic diversity among common reed populations from different salinity environments based on six microsatellite loci.**
(XLSX)

Table S3 **Mantel tests for the correlation between geographical and genetic distances.**
(XLSX)

Table S4 **Results of the spatial analysis using the program SAM, with significance level of (a) 5.05E-05; and (b) 5.05E-06 (corresponding to a 99% confidence level including Bonferroni correction).**
(XLSX)
