# Supplementary figures and images for: Spatial Genetic Structure in Natural Populations of Phragmites australis in a Mosaic of Saline Habitats in the Yellow River Delta, China
Source: PLoS One. 2012 Aug 16;7(8):e43334. doi: 10.1371/journal.pone.0043334 (PMC3420903; doi:10.1371/journal.pone.0043334)

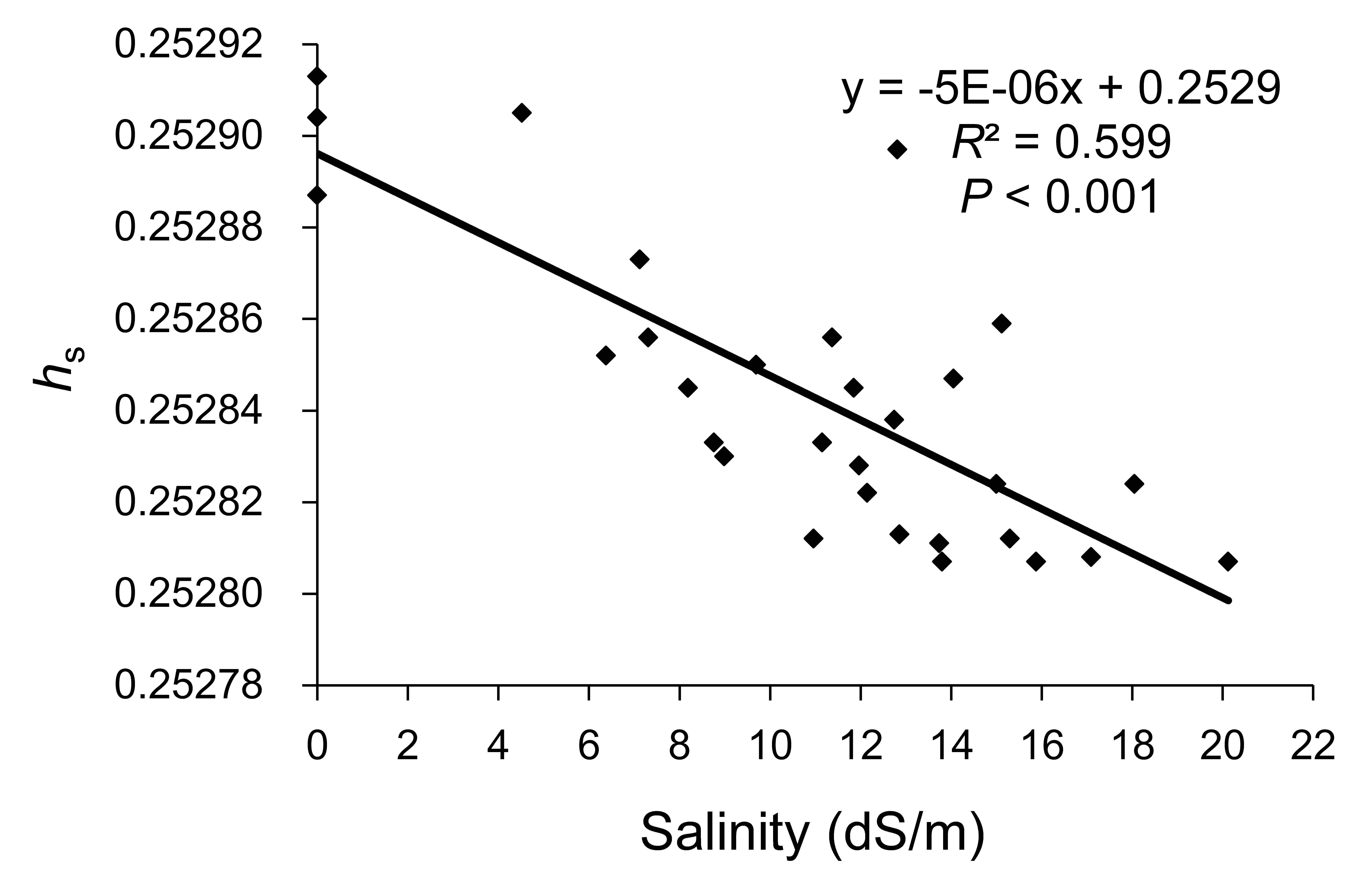

Supplement: Figure S1 — Correlation between soil salinity and the heterozygosity of each population ( h s) estimated with Hickory v1.1. (TIF) [file pone.0043334.s001.tif]
